# A Snapshot on MRSA Epidemiology in a Neonatal Intensive Care Unit Network, Palermo, Italy

**DOI:** 10.3389/fmicb.2016.00815

**Published:** 2016-05-27

**Authors:** Daniela M. Geraci, Mario Giuffrè, Celestino Bonura, Giorgio Graziano, Laura Saporito, Vincenzo Insinga, Grazia Rinaudo, Aurora Aleo, Davide Vecchio, Caterina Mammina, The NICU Surveillance Study Group, Amalia Ciofalo

**Affiliations:** ^1^Department of Sciences for Health Promotion and Mother–Child Care “G. D'Alessandro,” University of PalermoPalermo, Italy; ^2^Post-graduate Residency School in Hygiene and Preventive Medicine, University of PalermoPalermo, Italy; ^3^Post-graduate Residency School in Pediatrics, University of PalermoPalermo, Italy

**Keywords:** MRSA, active surveillance, network approach, molecular typing, NICu

## Abstract

**Objectives:** We performed a 1-year prospective surveillance study on MRSA colonization within the five NICUs of the metropolitan area of Palermo, Italy. The purpose of the study was to assess epidemiology of MRSA in NICU from a network perspective.

**Methods:** Transfer of patients between NICUs during 2014 was traced based on the annual hospital discharge records. In the period February 2014–January 2015, in the NICU B, at the University teaching hospital, nasal swabs from all infants were collected weekly, whereas in the other four NICUs (A, C, D, E) at 4 week-intervals of time. MRSA isolates were submitted to antibiotic susceptibility testing, SCC*mec* typing, PCR to detect *lukS-PV* and *lukF-PV* (*lukS/F-PV*) genes and the gene encoding the toxic shock syndrome toxin (TSST-1), multilocus variable number tandem repeat fingerprinting (MLVF), and multilocus sequence typing (MLST).

**Results:** In the period under study, 587 nasal swabs were obtained from NICU B, whereas 218, 180, 157, and 95 from NICUs A, C, D, and E, respectively. Two groups of NICUs at high prevalence and low prevalence of MRSA colonization were recognized. Overall, 113 isolates of MRSA were identified from 102 infants. Six MLVF types (A–F) were detected, with type C being subdivided into five subtypes. Five sequence types (STs) were found with ST22-IVa being the most frequent type in all NICUs. All the MRSA molecular subtypes, except for ST1-IVa, were identified in NICU B.

**Conclusions:** Our findings support the need to approach surveillance and infection control in NICU in a network perspective, prioritizing referral healthcare facilities.

## Introduction

Methicillin resistant *Staphylococcus aureus* (MRSA) is a frequent causal agent of serious healthcare related infections in neonatal intensive care units (NICUs; Carey and Long, 2010; Nelson and Gallagher, 2012). Host factors, such as prematurity and immaturity of the immune system, and some peculiarities of the NICU health care setting, such as prolonged hospitalization, exposure to invasive procedures and high frequency of handling by healthcare workers (HCWs), are acknowledged to promote colonization and infection by MRSA (Maraqa et al., 2011; Giuffrè et al., 2015). Overcrowding and understaffing can ultimately play a key role in driving cross-transmission and expand the MRSA reservoir (Andersen et al., 2002). In high endemic countries, MRSA colonization and infection are frequent in all healthcare facilities, but especially in those providing tertiary care, where selection and spread or influx of new clones of MRSA have higher probability to occur (Donker et al., 2010). Indeed, on a wide geo-temporal scale the emergence of major MRSA clones from previously circulating methicillin susceptible *S. aureus* (MSSA) clones, by acquisition of the methicillin resistance gene, has shaped the evolution of MRSA. Conversely, in a regional or local perspective, between patient transmission is believed to play a prominent role (Enright et al., 2002; Donker et al., 2010).

In recent years, the referral of patients between healthcare facilities has become the subject of many epidemiological studies focusing the spread dynamics of nosocomial pathogens, with special attention to multidrug resistant organisms (MDROs; Donker et al., 2012; Ciccolini et al., 2013, 2014). Indeed, patient and MDROs movement has been proven to stay behind many inter-institutional outbreaks, whose size and impact largely depend on the referral pattern of the site of the initial outbreak (Donker et al., 2012; Ciccolini et al., 2013, 2014). MRSA, being one of the most prevalent healthcare pathogens, has provided most useful insights about the epidemiological consequences of the inter-institutional patient transfer (Donker et al., 2012). Availability of accurate and discriminative typing techniques has undoubtedly facilitated these investigations (Azarian et al., 2015; Nelson et al., 2015).

NICUs are currently being organized into neonatal networks comprising a range of units providing different level and/or subspecialty care in a given regional setting. We here describe the findings of a 1-year prospective surveillance study on neonatal MRSA colonization within the five NICUs of the metropolitan area of Palermo, Italy. The purpose of the study was to provide an update on epidemiology of MRSA in NICU from a network perspective and, at the same time, to tentatively use MRSA molecular fingerprinting to assess the epidemiological effects of referral of patients within the NICUs in a high endemic area.

## Materials and methods

### Setting and design of the study

Since February 10, 2014 a network surveillance study is performed in five NICUs (NICUs A to E) in Palermo, Italy, including the III level NICU (NICU B) of the teaching University hospital AOUP “P. Giaccone.” This NICU is associated with the regional reference center for genetic diseases and a neonatal surgery unit. Consequently, the NICU admits a large proportion of neonates with malformations or complex conditions requiring surgical care (about 40%) as well as outborn infants transferred from other hospitals (about 35%). The annual admissions to this NICU average annually 250 infants. The metropolitan area of Palermo, Italy, includes four additional NICUs. Their main characteristics are summarized in Table 1.

**Table 1:** **Characteristics of the NICUs located in the metropolitan area of Palermo, Italy**.

**NICU**	**Intensive care room cot spaces (nr)**	**Intermediate care room cot spaces (nr)**	**Admissions in 2014**	**Subspecialty care**
A	10	14	429	Neurosurgery, Retinopathy of prematurity
B	8	8	256	Genetic diseases, Neonatal surgery
C	8	8	371	Perinatal hypoxic-ischemic encephalopathy
D	6	12	413	–
E	6	0	183	–

Transfer of patients between NICUs was traced by quantifying their movements based on the annual hospital discharge records deposited at the Regional Health Service Statistical Office. Only the first transfer was counted for each patient.

### Active surveillance program and case identification

In NICU B a surveillance program on MRSA colonization is in place since 2009 by universal nasal sampling (Giuffrè et al., 2015). Nasal swabs are collected on a weekly basis (each Monday) from all infants staying in NICU, regardless of their interval of time since admission. In NICUs A, C, D, and E an active surveillance program was initiated in February 2014. Nasal swabs were similarly collected the same day from all infants, but at 4 week-intervals of time. For the purposes of this study, each 4-week interval was defined as a month.

Inclusion criteria for all patients were admission between February 10, 2014 and January 11, 2015 (conventionally defined as a year), hospitalization for at least 48 h, and collection of at least one nasal swab. Colonization was defined as the isolation of MRSA from anterior nares without evidence of infection.

Staff training regarding active surveillance and infection control measures took place at the time of program implementation. Reports of the active surveillance program findings were regularly sent to the NICU staff each month. Patients with MRSA colonization or infection were cohorted, under standard contact precautions, but without dedicated personnel due to the staff shortage. No decolonization strategies were employed. Screening of HCWs and environmental monitoring for MRSA were not carried out.

The study protocol and data handling were approved by the ethical committee of the teaching University hospital AOUP “P. Giaccone,” Palermo, Italy. The study was performed according with the ethical standards established in the Declaration of Helsinki. Written informed consent was obtained from the parents or guardians of the neonates.

### Microbiological procedures and molecular typing

Surveillance specimens from the anterior nares of neonates were obtained with cotton swabs, immediately transferred to the laboratory and processed within 2 h. Nasal swabs were incubated overnight in Brain Hearth Infusion (BHI) broth (OXOID, Basingstoke, UK) and then plated onto mannitol salt agar (OXOID), incubated in air at 35°C and examined at 24 and 48 h. Presumptive *S. aureus* isolates were identified according to standard methods. MRSA isolates were searched for by colony screening onto oxacillin agar (Mueller-Hinton with oxacillin 6 mg/L) and confirmed by the cefoxitin disk diffusion test and a *mec*A PCR (Boşgelmez-Tinaz et al., 2006).

All isolates from MRSA colonized patients were submitted to antibiotic susceptibility testing and genotyping. Antibacterial drug susceptibility was routinely performed using the disk diffusion method by determining the susceptibility of each isolate to erythromycin, clindamycin, sulfamethoxazole-trimethoprim, tetracycline, ciprofloxacin, gentamicin, tobramycin, linezolid, rifampicin, vancomycin, and teicoplanin. *S. aureus* ATCC 25923 was used as the quality control strain. Results were interpreted using the European Committee on Antimicrobial Susceptibility Testing (EUCAST) clinical breakpoints (http://www.eucast.org/fileadmin/src/media/PDFs/EUCAST_files/Breakpoint_tables/).

Staphylococcal cassette chromosome (SCC) *mec* was typed by the multiplex PCR method described by Milheiriço et al. (2007). PCR was also performed with primers designed to detect *lukS-PV* and *lukF-PV* (*lukS/F-PV*) genes, which encode Panton-Valentine leukocidin (PVL), and the gene encoding the toxic shock syndrome toxin (TSST-1), as described previously (Mehrotra et al., 2000; Zhang et al., 2008).

Genotypic characterization of the MRSA isolates was performed by multilocus variable number tandem repeat fingerprinting (MLVF; Karynski et al., 2008). Gel images were stored as TIFF files. Banding patterns were analyzed both visually and by using Bionumerics version 5.10 (Applied Maths, Sint-Martens-Latem, Belgium). The MLVF patterns consist of five to seven bands. A difference of five or more bands between the patterns was interpreted as a different type, and each type was attributed with a capital letter. In the event of patterns differing by less than five bands, they were categorized as subtypes of a type, and each subtype identified by an additional numeric suffix. Only indistinguishable patterns were attributed with the same subtype. After a preliminary visual checking, the MLVF patterns were analyzed using Bionumerics with the following parameters: optimization 0.5% and position tolerance 1.25%. Pairwise similarity coefficients were calculated using the Dice formula, and dendrograms were created using the unweighted pair-group method using geometric averages (UPGMA). The similarity cutoff value between the MLVF patterns was set up at the level of 70%.

Representative strains of all the different MLVF patterns were analyzed by multilocus sequence typing (MLST). The MLST allelic profiles and sequence types were assigned by submission to the *S. aureus* MLST database (www.saureus.mlst.net).

### Statistical analysis

Monthly prevalence of MRSA colonization was assessed using the number of colonized infants at the time of nasal sampling for the NICUs A, C, D, and E and the sum of the number of colonized infants in the 4 days of sampling for NICU B. The number of infants staying in NICU at the time of nasal sampling and the total number of infants in the 4 days of sampling were used as denominator, respectively, for NICUs A, C, D, and E and for NICU B. Annual medians and interquartile ranges (IQRs) were also estimated.

## Results

**Figure 1:**
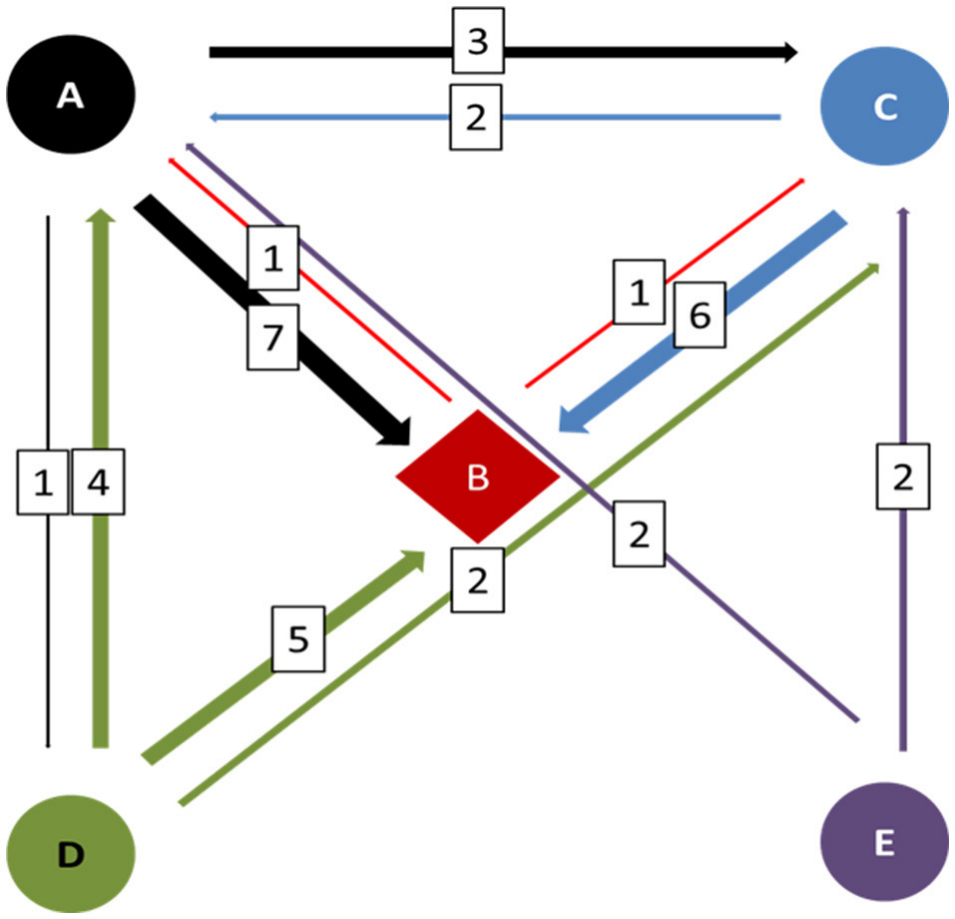
**Diagrammatic representation of the NICU referral network in the metropolitan area of Palermo, Italy, in 2014**. The thickness of the arrows is roughly proportional to the number of infants (in the squares) who were referred between NICUs. Different colors indicate the directionality, identifying the referring NICU.

The number of admissions in the five NICUs under study in the year 2014 is shown in Table 1. The structure of the NICU referral network and the movements between NICUs are schematically represented in Figure 1. Within this network, NICU B exhibits the highest degree of connectivity, whereas NICUs A and C assume an intermediate position and NICUs D and E work as referring units only. The patient flow through the network was thus prominently directed toward NICU B, and to a lower extent toward NICUs A and C, both providing specific subspecialty care. Because only the first transfer was considered, but infants with complex conditions and prolonged NICU stays can move through the NICU network several times with different directionality, the referral pattern could be substantially denser compared with the number of shared patients.

**Figure 2:**
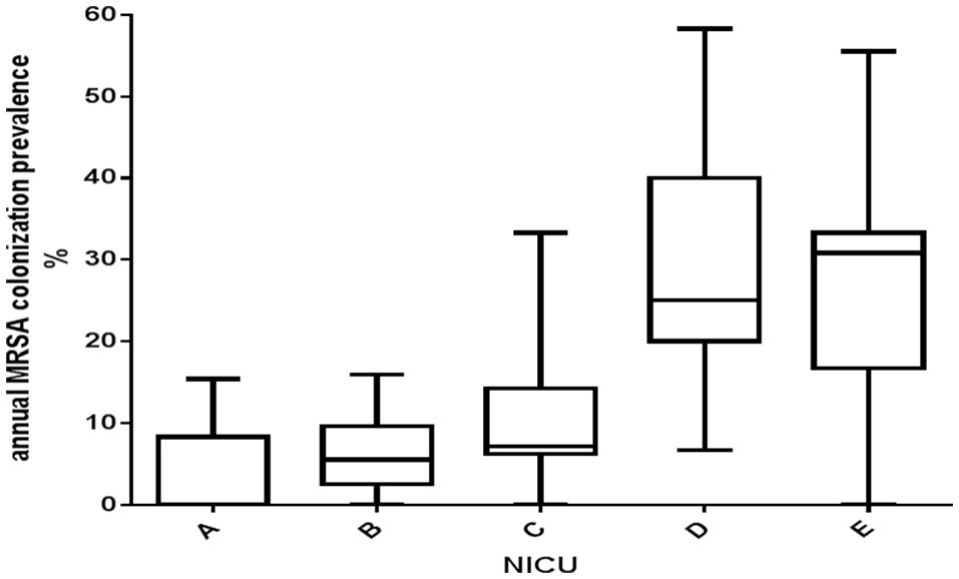
**Boxes and whiskers representation of the annual average prevalence of MRSA colonization in the five NICUs under study, Palermo, Italy, 2014**. The median is shown by the dark horizontal line. The 25th and 75th percentiles comprise the *boxes*; *whiskers* extend to the minimum and maximum values.

In the period under study, 587 nasal swabs were obtained from NICU B, whereas 218, 180, 157, and 95 were obtained from NICUs A, C, D, and E, respectively. The median prevalence of MRSA colonization in the five NICUs in the interval of time under consideration is illustrated in Figure 2. Two groups of NICUs, high prevalence (D and E) and low prevalence (A, B, and C), could be recognized.

**Figure 3:**
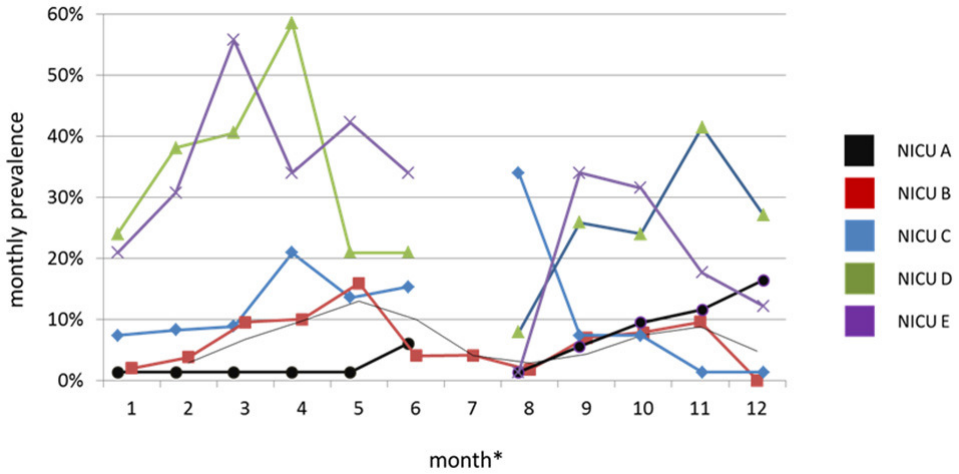
**Monthly prevalence of MRSA colonization in the NICUs under surveillance, Palermo, Italy, 2014**. In August, the active surveillance culture (ACS) program in the NICUs A, C, D and E was interrupted.*Month is a 4-week interval of time.

During the period of surveillance, the MRSA colonization prevalence substantially differed between NICUs and fluctuated widely within each NICU (Figure 3). A general decreasing trend was observed through all NICUs except for NICU A, which consistently exhibited the lowest prevalence during the entire period, except for the last 2 months.

### Infection control and prevention policies

A strengthening of the routine infection control policies was adopted in all NICUs. They included: a revision of the invasive devices management, adoption of contact precautions and cohorting of infants colonized or infected by MRSA, use of dedicated equipment and intensified environmental sanitation. Cleaning policies included post-discharge cot terminal cleaning in the disinfection room, irrespective of the MRSA carriage status of the occupant. Moreover, attention was dedicated to prevent overcrowding and relative understaffing and minimize length of hospital stay. No neonates were treated with mupirocin for decolonization. MRSA screening of HCWs was not carried out in any NICU. Cyclic training sessions of the NICUs staff were planned. Inter-NICU communication regarding MRSA colonization and infection and individual unit's management of shared infants was implemented. Infection prevention and control was identified as an audit of importance for all the NICUs and supportive for the network as a whole in regard to transfers and patient pathways and good practice.

### Strain characterization

During the period under study, a total of 113 isolates of MRSA were identified from 102 infants. Their antibacterial drug resistance patterns and molecular epidemiological characteristics are summarized in Table 2.

**Table 2:** **Antimicrobial drug resistance patterns and molecular types of the MRSA isolates identified from infants staying in the five NICUs of Palermo, Italy, 2014**.

**NICU**	**ST/SCC*mec***	MLVF type/subtype^*^	**Resistance pattern**	**nr. isolates**	**nr. patients**
A	1/IVa	F	TE	1	1
	22/IVa	C1	Susceptible	6	6
	22/IVa	C2	Susceptible	1	1
	22/IVa	C3	TE	1	1
B	5/IVg	A	GN	3	2
	22/IVa	C1	Susceptible	21	15
	22/IVa	C2	Susceptible	1	1
	22/IVa	C2	TE	2	2
	22/IVa	C3	Susceptible	1	1
	80/IVc	B	TE	2	2
	217/IVh	E	Susceptible	1	1
C	22/IVa	C1	Susceptible	5	5
	22/IVa	C2	Susceptible	7	7
D	5/IVg	A	GN	10	10
	22/IVa	C1	Susceptible	6	6
	22/IVa	C2	Susceptible	3	3
	22/IVa	C2	TE	12	11
	22/IVa	C4	Susceptible	1	1
	22/IVa	C5	Susceptible	1	1
	217/IVh	E	Susceptible	4	4
E	1/IVa	D	TE	3	1
	22/IVa	C1	Susceptible	6	5
	22/IVa	C2	Susceptible	16	16

ST, sequence type; SCCmec, Staphylococcal Cassette Chromosome mec; MLVF, Multiple Locus Variable Number of Tandem Repeat Fingerprinting; TE, tetracycline; GN, gentamicin

* MLVF types are identified by capital letters and subtypes by capital letters followed by Arabic numbers.

**Figure 4:**
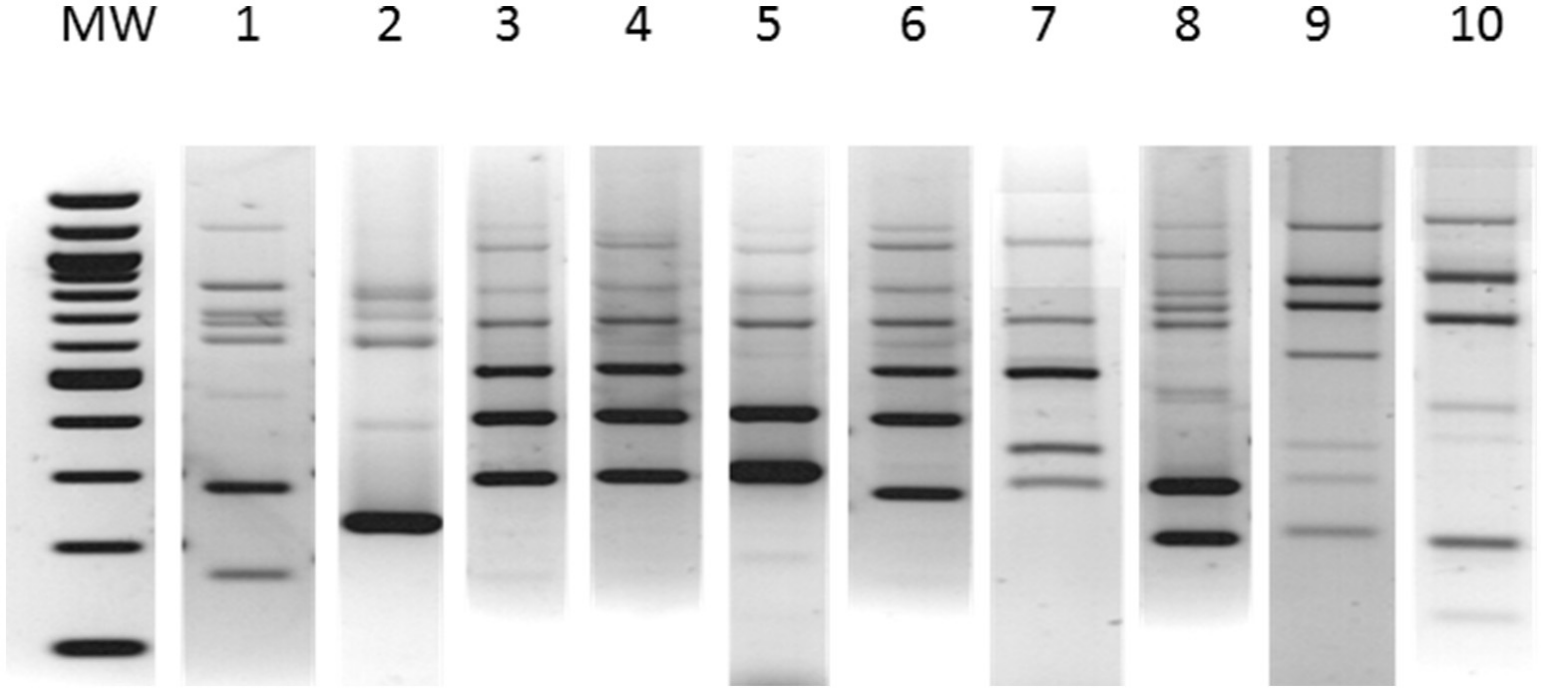
**Multilocus variable number tandem repeat fingerprinting (MLVF) patterns identified in the study**. Lanes: MW, 100 bp DNA ladder; 1 to 10, MLVF patterns A, B, C1, C2, C3, C4, C5, D, E, F.

Seventy-nine of 113 isolates (69.9%) were fully susceptible to the panel of antimicrobial drugs tested. Twenty-one isolates (18.6%) proved to be resistant to tetracycline and 13 (11.5%) to gentamicin. Six MLVF types named A to F were identified, with type C being subdivided into five different subtypes (Figure 4). Type C accounted for 90 isolates (79.6%) and subtype C1 for 44 out of 90 isolates (48.9%). Overall, five STs were identified. ST22-IVa was the most frequent type and proved to be invariably linked with the MLVF type C. A close relatedness was generally apparent between MLVF types and STs, except for the four ST1-IVa isolates which were attributed with two different MLVF types. Moreover, resistance to gentamicin was strictly associated with the ST5-IVg, MLVF type A isolates, whereas resistance to tetracycline was more erratic, being exhibited by all ST1-IVa and ST80-IV isolates and some ST22-IVa–MLVF subtypes C2 and C3 isolates.

All ST22-IVa isolates were *tst1*-positive. The two ST80-IVc isolates tested positive for PVL and negative for TSST-1 genes. All the remaining isolates were negative for both gene sequences.

### Colonization cases in the five NICUs based upon MRSA typing

In all NICUS infants were detected who tested positive for ST22-IVa subtypes C1 and C2. MRSA isolates characterized as ST22-IVa, subtype MLVF C1 and fully susceptible were predominant in all NICUs, except NICU D. Conversely, MRSA isolates ST22-IVa, subtype MLVF C2, and tetracycline resistant appeared to be endemic in NICU D, being carried by 11 different neonates during the surveillance period. A further interesting feature was the detection of gentamicin resistant MRSA isolates ST5-IVg with an apparently endemic pattern in NICU D, but also as a sporadic finding in NICU B. Four infants staying in NICU D were colonized by a ST217-IVh MRSA strain which was also found in one patient in NICU B. It is noteworthy the detection of two neonates in NICU B carrying a ST80-IVc strain harboring genes for PVL. The index case was an infant born to Tunisian parents.

## Discussion

We previously reported about MRSA colonization in the III level NICU of the University Hospital of Palermo, Italy, where a proportion as high as 22.8% of the admitted infants had a nasal surveillance culture growing MRSA during the 4-year period June 2009–June 2013 (Giuffrè et al., 2015). The prominent presence of a strain characterized as *tst*1 positive, UK-EMRSA-15/“Middle Eastern Variant,” which had been prior documented, was confirmed (Geraci et al., 2014a). The same clone was also detected in eight out of ten MRSA positive healthy preschool children during a survey conducted in 2013 on 500 children attending the municipal daycare centers in the same town (Geraci et al., 2014b).

Here we performed a surveillance prospective study on MRSA colonization in NICU with a network approach, targeting the five NICUs located in the metropolitan area of Palermo, Italy, and connected via the referred patients. Due to its endemicity in our geographic setting, its clonal heterogeneity and the availability of reliable and discriminative typing technique, MRSA is a microrganism especially suited to trace movements between community and NICUs and between different NICUs.

Some interesting findings can be emphasized. The first is the evidence of different prevalences of MRSA colonization among NICUs, with those providing subspecialty care showing lower rates compared with those that do not. This can be speculatively attributed to more stringent infection control practices in the first ones, driven by the need to safely deliver sophisticated care practices to fragile neonates. It should be also considered that NICU D, the MRSA top-prevalent NICU, is associated with the higher volume of births per annum in the maternity ward. In an endemic setting, such as ours, where MRSA is spreading in both healthcare facilities and community, this peculiar feature could have favored the influx of MRSA strains into the NICU (Jimenez-Truque et al., 2012; Lazenby et al., 2012). It is also noteworthy that the referral III level NICU B had comparatively low prevalences through the entire period of observation. In this NICU weekly ASCs, which are routinely carried out since 2009, are integrated into a multifaceted infection control program. This has been previously proved to be associated to a containment of MRSA cross-transmission and could likely explain the better performance of NICU B (Giuffrè et al., 2015).

The second significant epidemiological feature is the further evidence of the major role in our geographic setting of the *tst*1 positive, UK-EMRSA-15/“Middle Eastern Variant” strain, known as the “Gaza strain” (Biber et al., 2012; Al Laham et al., 2015). Cross-transmission has likely contributed to the NICU clusters of patients carrying isolates belonging to the different MLVF subtypes. It is also interesting to observe that, except for the sporadic C4 and C5 subtypes, the three most prevalent subtypes, C1, C2, and C3, were all detected in NICU B. Because of the documented presence of such a strain in the community, its apparent endemicity can be interpreted as the result of an import from the community or a trade within NICUs via referred patients or both. Unfortunately, the design of this study does not allow for an unambiguous answer. Nevertheless, in our experience, MLVF confirmed to be a quick and easy method to differentiate MRSA isolates within the CC22 complex. High discriminatory power coupled with simplicity and low costs make MLVF especially suitable to trace transmission routes and investigate MRSA local epidemiology (Karynski et al., 2008).

Of further interest are the cluster of gentamicin resistant ST5-IVg and the smaller cluster of ST217-IVh isolates, both occurring in NICU D. CC5-MRSA-IV strains, colloquially described as the “Paediatric Clone” have gained pandemic diffusion and significant clinical relevance (Enright et al., 2002). Conversely, ST217, a single-locus variant of EMRSA-15 within the CC22, has been infrequently reported in literature, and once only in Italy from the nasal swabs of a 5-year-old boy and his parents (Vignaroli et al., 2014). Recently, emergence of this clone in the community has been reported in India (Bouchiat et al., 2015). Both these MRSA clones proved to be present with a limited number of isolates in NICU B, as the result of a possible spillover into this referral NICU.

Finally, two isolates of PVL positive, “European clone” ST80-IVc were detected from two neonates in NICU B, with the index case being a Tunisian children. Only a secondary case was recorded due to the prompt alert to the NICU staff and the infection control measures being routinely in place.

ST80/IV has consistently been recognized as the predominant PVL-positive MRSA clone in North African and Middle East countries (Harastani and Tokajian, 2014). In some European countries ST80-MRSA has been sporadically detected and in many cases associated with travel histories in Mediterranean and Middle East countries (Harastani and Tokajian, 2014). A recent study has provided solid evidence that the clonal spread of MRSA ST80-IV has been boosted by the increased transnational movements (Stegger et al., 2014).

The epidemiological snapshot of MRSA distribution in the five NICUs underlines the presence of all MRSA types and subtypes, except for those at lowest prevalence, such as ST1 and ST22 MLVF subtypes C4 and C5, in the referral NICU B as a likely consequence of its more dense referral pattern with the other NICUs.

This study has some limitations. The ASC program was not identical in all NICUs, with only infants in NICU B being sampled weekly. Consequently, the results are not quite comparable both in terms of prevalences and representativeness of MRSA isolates. Indeed, the prevalence calculated in NICUs with a low cot space number could have been easily misestimated by a monthly point-prevalence measure. Conversely, the monthly prevalences of NICU B were more solid and, due to the more frequent sampling, all MRSA types/subtypes, including those with low prevalence, had a higher probability of detection. Moreover, movements of infants within NICUs were not traced at the individual level, which could have made it possible to incontestably confirm the trade of MRSA isolates within the NICU network.

In accordance with the purpose of the study, we performed a preliminary study to describe the epidemiology of MRSA in the NICU network of a large metropolitan area, previously known to be high-endemic. At the same time we tried to palpably depict the connection pattern within NICUs and its epidemiological impact, using the consolidated molecular epidemiological markers of MRSA. Our findings support the need to approach surveillance and infection control in a network perspective, prioritizing referral healthcare facilities. Further studies are necessary to consolidate our results.

## Author contributions

DG, MG, and CM were responsible for the conception and design of the study. DG, GG, VI, GR, LS, and DV were involved in the acquisition and analysis of data; DG, CB, and AA were in charge of molecular typing of MRSA isolates; MG and CM interpreted the data and drafted article; all authors revised it critically and approved the version to be submitted.

## Funding

This work was funded by institutional resources.

### Conflict of interest statement

The authors declare that the research was conducted in the absence of any commercial or financial relationships that could be construed as a potential conflict of interest.
